# Peripheral Blood Mononuclear Cells Derived from Grand Multigravidae Display a Distinct Cytokine Profile in Response to *P. falciparum* Infected Erythrocytes

**DOI:** 10.1371/journal.pone.0086160

**Published:** 2014-01-22

**Authors:** Louise E. Ludlow, Wina Hasang, Alexandra J. Umbers, Emily K. Forbes, Maria Ome, Holger W. Unger, Ivo Mueller, Peter M. Siba, Anthony Jaworowski, Stephen J. Rogerson

**Affiliations:** 1 Department of Medicine (RMH), University of Melbourne, Post Office Royal Melbourne Hospital, Melbourne, Victoria, Australia; 2 Victorian Infectious Diseases Service, Royal Melbourne Hospital, Grattan Street, Parkville, Victoria, Australia; 3 Papua New Guinea Institute of Medical Research, Vector Borne Disease Unit, Madang, PNG; 4 The Walter and Eliza Hall Institute of Medical Research, Melbourne, Australia; 5 Barcelona Centre for International Health Research (CRESIB), Hospital Clínic-Universitat de Barcelona, Barcelona, Spain; 6 Centre for Biomedical Research, Burnet Institute, Melbourne, Victoria, Australia; 7 Department of Infectious Diseases, Monash University, Victoria, Australia; 8 Department of Immunology, Monash University, Victoria, Australia; Institut de Recherche pour le Développement, France

## Abstract

Immunopathology of placental malaria is most significant in women in their first pregnancy especially in endemic areas, due to a lack of protective immunity to *Plasmodium falciparum*, which is acquired in successive pregnancies. In some studies (but not all), grand multigravidae (defined as 5 or more pregnancies, G5–7) are more susceptible to poor birth outcomes associated with malaria compared to earlier gravidities. By comparing peripheral cellular responses in primigravidae (G1), women in their second to fourth pregnancy (G2–4) and grand multigravidae we sought to identify key components of the dysregulated immune response. PBMC were exposed to CS2-infected erythrocytes (IE) opsonised with autologous plasma or unopsonised IE, and cytokine and chemokine secretion was measured. Higher levels of opsonising antibody were present in plasma derived from multigravid compared to primigravid women. Significant differences in the levels of cytokines and chemokines secreted in response to IE were observed. Less IL-10, IL-1β, IL-6 and TNF but more CXCL8, CCL8, IFNγ and CXCL10 were detected in G5–7 compared to G2–4 women. Our study provides fresh insight into the modulation of peripheral blood cell function and effects on the balance between host protection and immunopathology during placental malaria infection.

## Introduction

Placental malaria due to *Plasmodium falciparum* infection is characterised by the accumulation of infected erythrocytes (IE) in the intervillous spaces of the placenta. Immunopathology of pregnancy-specific malaria is largely confined to women in their first and second pregnancy due to a lack of protective immunity, and results in increased risk of severe anemia and low birth weight (LBW), especially in highly endemic areas [1]. These complications are associated with monocyte and macrophage accumulation in the maternal intervillous circulation of the placenta, termed intervillositis [2], and with increased placental blood TNF [3]. As a result of phagocytic uptake of IE, placental monocytes and macrophages from malaria-infected pregnant women contain the bioactive pigment haemozoin, which is the undigestable product of haemoglobin metabolism by the intra-erythrocytic parasite. IE phagocytic clearance represents an important mechanism of controlling blood trophozoite-stage parasites and is enhanced by antibody opsonisation [4].

The variant surface antigen VAR2CSA displayed on the IE surface mediates binding to chondroitin sulphate A (CSA) expressed on the placental syncytiotrophoblast and in placental blood, representing the principal target for opsonising antibody. Antibodies against VAR2CSA block placental sequestration and opsonise IE for phagocytic uptake. These antibodies develop with gravidity, and are associated with protection against LBW and severe maternal anaemia [5]–[8]. In successive pregnancies, women progressively acquire antibody-mediated immunity to VAR2CSA, and both prevalence and density of *P. falciparum* infections decline [1], [9]–[12]. In some studies, grand multigravidae (fifth or subsequent pregnancies) showed an increased risk of malaria suggesting they lose some of their acquired immunity compared to earlier gravidities, and malaria chemoprophylaxis significantly increased the birth weights of their babies (but did not benefit women in second to fourth pregnancy) [10]–[12]. Waning immunity was mooted as an explanation, but no confirmatory studies were reported.

Successful pregnancy outcome in the context of malaria infection depends on appropriate regulation of pro-inflammatory and anti-inflammatory cytokines to achieve equilibrium between immunity and the risk of inflammation-associated severe malaria. Studies of malaria-infected placentas confirm that pro-inflammatory cytokines and β chemokines are secreted by intervillous macrophages and monocytes in response to IE [13]. Cytokines elevated in placental malaria include TNF, IFNγ, IL-1β, IL-10, CXCL8, CXCL10 and CCL3 [3], [13]–[17]. Elevated TNF levels have been associated with LBW and anemia [3], [17] while IFNγ is thought to be a critical factor in protection against placental malaria [14]. Increased production of β chemokines including CXCL8 may be an important trigger for monocyte accumulation in the placenta [13]. The alteration in cytokine balance in the setting of maternal malaria infection is important for clearance of IE from the placenta, but is also associated with maternal anaemia, premature delivery and spontaneous abortion [3], [14], [15], [18]. The immune response to malaria is dominated by Th-1 cytokines and β chemokines which, while limiting parasitaemia by promoting phagocytosis of IE, may also contribute to placental immunopathology [18], [19]. By comparing peripheral cellular responses across different gravidities in response to IE we aimed to characterize key components of the dysregulated immune response.

We hypothesised that differences in cytokine responses between multigravidae and grand multigravidae might explain the greater susceptibility of grand multigravidae to malaria. Peripheral blood mononuclear cells (PBMC) obtained from primigravidae (G1), women in their second to fourth pregnancy (G2–4) or their fifth or later pregnancies (G5–7) were exposed to unopsonised IE and IE opsonised with heat inactivated (HI) autologous plasma, and culture supernatants were collected for measurement of cytokines and chemokines. Using a phagocytosis assay to measure opsonising antibody levels, higher levels of opsonising antibody were present in plasma derived from multigravid compared to primigravid women. PBMC from G5–7 women secreted lower levels of IL-10, IL-1β, IL-6 and TNF but higher levels of CXCL8, CCL8, IFNγ and CXCL10 in response to IE than gravida 2–4 women. Our study sheds new light on the role of peripheral blood cells derived from pregnant women in the immune response to malaria.

## Materials and Methods

### Ethics Statement

The study was approved by the Institutional Review Board of the PNG Institute of Medical Research, the PNG Medical Research Advisory Committee (MRAC 10.50, parent study MRAC 08.01) and Melbourne Health Human Research Ethics Committee, Melbourne. Witnessed, written informed consent was provided by all participants.

### Study site and participants

Peripheral blood and plasma samples were collected between 2009–2011 as part of studies on malaria prevention in pregnancy in Madang Province, PNG. Geographic location of the study site, annual rainfall and malaria transmission have been previously described [20]. Samples were collected from women at enrolment (14–26 weeks) before initiation of antimalarial therapy. Peripheral blood smears were tested for malaria by microscopy by at least two qualified microscopists. Thick films were examined for 200 high-powered fields before being declared infection-negative. Five women were subsequently found to have *P falciparum* infection by PCR only (three gravida 1, one each gravida 4 and gravida 6). Exclusion of these women did not significantly affect the results. Women then received insecticide-impregnated bed nets and sulphadoxine-pyrimethamine (SP) and chloroquine, or repeat doses of SP and azithromycin. Only PBMC from pregnant women without current malaria infection were analysed in this study, and consisted of PBMC obtained from 33 primigravid and 34 multigravid women. The multigravid group was further divided into women in their second to fourth pregnancy (G2–4, n = 16) or five or more pregnancies (defined as grand multigravidae, G5–7, n = 18). Maternal clinical characteristics are outlined in Table 1.

**Table 1:** Maternal characteristics of the study group.

	G1	G2–4	G5–7	p (ANOVA)
Maternal age at enrolment, years	20.8 (2.7)	27.4 (5.2)	33.5 (3.6)	<.0001
Maternal haemoglobin level, g/dL	9.75 (1.6)	9.6 (1.3)	10.0 (1.6)	0.79
Recruitment gestational age, weeks (by fundal height)	21.3 (4.4)	21.2 (4.0)	20.6 (3.6)	0.65

Data are mean (SD).

### Isolation of Peripheral Blood Mononuclear Cells and plasma

Sodium heparin vacutainers containing venous blood were centrifuged (1,800×*g*, 10 min) and plasma collected and stored at −80°C. Blood cells were resuspended in RPMI 1640 (Gibco) at room temperature and transferred to a 15 mL tube before PBMC isolation by density gradient centrifugation using Histopaque (Sigma). Plasma was clarified (16,100×*g*, 2 min) and inactivated at 57°C for 45 min.

### Culture and purification of IE

The laboratory-adapted *P. falciparum* line CS2 resembles placental-type isolates based upon VAR2CSA expression, binding to CSA and recognition by serum in a pregnancy and gravidity-specific manner. Trophozoite-stage IE were purified to 92–95% by density gradient centrifugation as described [21], frozen at 1.5×10^8^ per vial using glycerolyte (twice the volume of packed IE) and thawed gently when required as previously described [22].

### Phagocytosis assay using THP-1 cells

Phagocytosis assay to measure levels of opsonising antibody was performed using the non-adherent human monocyte cell line THP-1 maintained as described [23]. Purified trophozoite-stage CS2 IE were stained with 10 µg/mL ethidium bromide for 30 min at room temperature, washed four times in RPMI-HEPES, opsonised with 10% plasma for 1 h at room temperature and washed as before. THP-1 cells (2.5×10^4^) were added to opsonised IE (2.5×10^5^, a 1∶10 ratio) in 96-well plates, and incubated at 37°C for 40 min. Uningested IE were removed using FACS Lysing Solution (BD Biosciences) as described [23] and THP-1 cells were fixed in cold 2% paraformaldehyde in PBS before acquisition using a HyperCyt® CyAn flow cytometer (Beckman Coulter).

### PBMC elicitation assay

IE were left unopsonised or opsonised with 9% HI autologous plasma for 30 min at room temperature as described previously [21] [24]. IE were diluted in PBS to 1×10^8^ per mL and added immediately at a ratio of 3 per PBMC (6×10^5^ or 6 μL).

Vials of PBMC were thawed rapidly in a 37°C water bath, washed twice in warm cell culture medium and cell number and viability were determined in a haemocytometer using trypan blue exclusion. Cell viability was always 80–95%. PBMC were resuspended at 2×10^6^ per mL and 100 μL was added to U-bottom 96-well plates (Falcon MICROTEST™ Catalogue Number: 353077, Becton Dickinson Labware) and cells were rested for 1 h at 37°C. Unopsonised IE, opsonised IE or an equivalent number of uninfected erythrocytes (E) were added to PBMC at a 3∶1 ratio and 1% phytohaemagglutinin (PHA, Life Technologies) was used as a viability control. After 48 h incubation (established using a time course pilot study), culture supernatants from replicate wells were pooled and clarified (9,000×*g*, 2 min, RT), aliquoted and stored at −80°C. Supernatants harvested from resting PBMC and PBMC exposed to E or PHA were assessed for IL-6 secretion using ELISA (MABTECH) prior to completion of the multi-plex assay. Ten of 83 PBMC were considered to have been contaminated during purification and were excluded from further analysis (if IL-6 response to media/IL-6 response to PHA or E) ×100% ≥75% (criterion defined by Julia Cutts, Schofield laboratory, WEHI). A further 2 samples were unresponsive to PHA and 3 yielded insufficient cell numbers and were therefore excluded. A range of cytokines and chemokines in culture supernatant were tested using multi-plex assay (Custom design, Bio-Rad) including IL-1β, IL-1Ra, IL-6, IL-10, IFNγ, CXCL10, CCL3 and TNF. CXCL8 and CCL8 were measured by ELISA (R&D Systems).

### Data analysis and statistical treatment

Resting PBMC cytokine production was subtracted from IE-induced cytokine to eliminate the contribution of constitutive production and data is presented on a log_10_ scale due to high inter-sample variability. Where constitutive cytokine production exceeded that of IE-induced cytokine a value of zero was assigned; this occurred for IL-1Ra (2 samples), CXCL8 (5 samples) and CXCL10 (14 samples). For CCL3, nine samples out of 68 were higher than the standard curve and were assigned the highest standard curve value of 21,878 pg/mL. Comparison between three gravidity groups (G1, G2–4, G5–7) was performed using a non-parametric One Way ANOVA: Kruskal-Wallis test and the Dunn's Multiple Comparison post-test. For comparison between two paired groups (e.g. G1 IE and G1 IE-IgG) a non-parametric Wilcoxon matched-pairs signed rank test was performed. Statistical analysis was carried out using Prism 5.0 software (GraphPad Software) and significance was defined as a probability value below 0.05.

## Results

### Clinical characteristics of the study group

A significant difference in maternal age at enrolment of G1 (mean (SD) 20.8 (2.7); years) compared to G2–4 (27.4 (5.2) and G5–7 (33.5 (3.6) (Table 1) was observed. Haemoglobin levels and recruitment gestational age did not differ. Bed net use was not significantly different and as the samples were collected at enrolment no difference in treatment with SP and chloroquine, or repeat doses of SP and azithromycin was possible.

### Higher levels of opsonising antibody are present in plasma derived from multigravid compared to primigravid women

**Figure 1:**
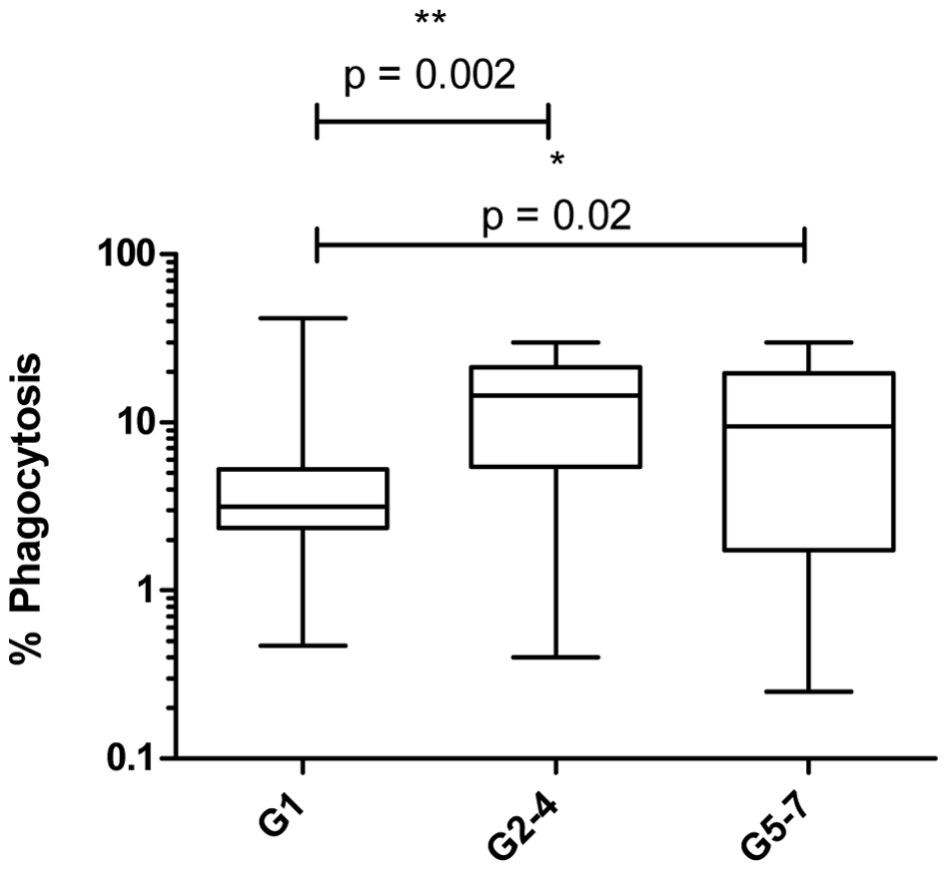
Higher levels of opsonising antibody are present in plasma derived from G2–4 and G5–7 compared to primigravid (G1) women. Phagocytosis assay was performed using 2×10^4^ THP-1 cells incubated for 40 min with 2.5×10^5^ CS2 IE opsonised with HI plasma derived from 33 G1, 16 G2–4 and 18 G5–7 women. Data are represented as percentage of THP-1 cells that have ingested IE (% Phagocytosis). Background non-specific phagocytosis observed with sera from naïve controls was subtracted and data are the average of samples analyzed in duplicate. Data are presented on a log_10_ scale, bars represent median and interquartile ranges, whiskers represent 95% CI. Statistical analysis was carried out using a non-parametric Mann Whitney test.

Levels of opsonising antibody were assessed using a THP-1 phagocytosis assay in which CS2 IE were opsonised with HI plasma derived from 33 primigravid and 34 multigravid women who were further divided into secundigravidae to gravidae 4 (G2–4) and grand multigravidae (G5–7). Significantly higher levels of opsonising antibody were detected in plasma obtained from G2–4 (p = 0.002) and G5–7 (p = 0.02) compared to G1 (Figure 1). There was no significant difference in opsonising antibody between G2–4 and G5–7 (p = 0.64).

### Constitutive cytokine secretion and comparison of opsonised versus unopsonised IE

We examined constitutive cytokine secretion by resting PBMC not exposed to IE and for most cytokines analysed no significant difference was recorded (data not shown). Less IL-10 (median (IQR) 3 (2–6) versus 6 (3–14) pg/mL; p<0.05) was detected in resting PBMC derived from G1 compared to G5–7.

Opsonisation of IE resulted in increased TNF, IL-1Ra and CCL3 secretion in G1, G2–4 and G5–7 (Table 2). Following opsonisation, IL-1β levels were increased in G1 and G5–7 whereas IL-6 levels were increased in G1 only. Opsonisation resulted in higher IL-10 in G1 but lower levels in G5-7. There was lower CXCL10 secretion in all groups and lower IFNγ secretion in G1 in response to opsonised IE. Secretion of CXCL8 and CCL8 did not differ between PBMC exposed to opsonised and unopsonised IE (Table 2).

**Table 2:** Comparison of cytokines and chemokines secreted by PBMC exposed to opsonised and unopsonised IE.

	G1	G2–4	G5–7
**IL-10**	1.2 (0.9–1.6) *	ns	0.6 (0.4–0.8) *
**IL-1β**	1.3 (1.2–2.4) ***	ns	1.6 (1.1–3.0) *
**IL-6**	1.1 (1.0–1.4) **	ns	ns
**TNF**	1.4 (0.9–1.7) **	1.5 (1.2–2.3) **	1.4 (1.1–1.8) **
**CXCL8**	ns	ns	ns
**CCL8**	ns	ns	ns
**IFNγ**	0.7 (0.6–1.1) *	ns	ns
**CXCL10**	0.6 (0.2–0.8) ***	0.7 (0.3–0.9) ****	0.5 (0.4–0.7) ***
**IL-1Ra**	1.6 (1.2–2.1) ****	1.4 (1.3–1.7) **	1.5 (1.1–2.1) ***
**CCL3**	1.6 (1.0–2.8) **	1.2 (1.0–1.4) *	1.9 (1.1–2.6) *

Ratios were calculated by dividing the cytokine or chemokine secretion detected in response to opsonised IE by that in response to unopsonised IE. Values represent median and IQR: only values which are significantly different to 1.0 are shown. For comparison between two paired groups a non-parametric Wilcoxon matched-pairs signed rank test was performed. * p<0.05, ** p<0.01, *** p<0.001, **** p<0.0001.

Not significant (ns).

### PBMC derived from G5-7 women secrete lower levels of IL-10 and IL-1β

**Figure 2:**
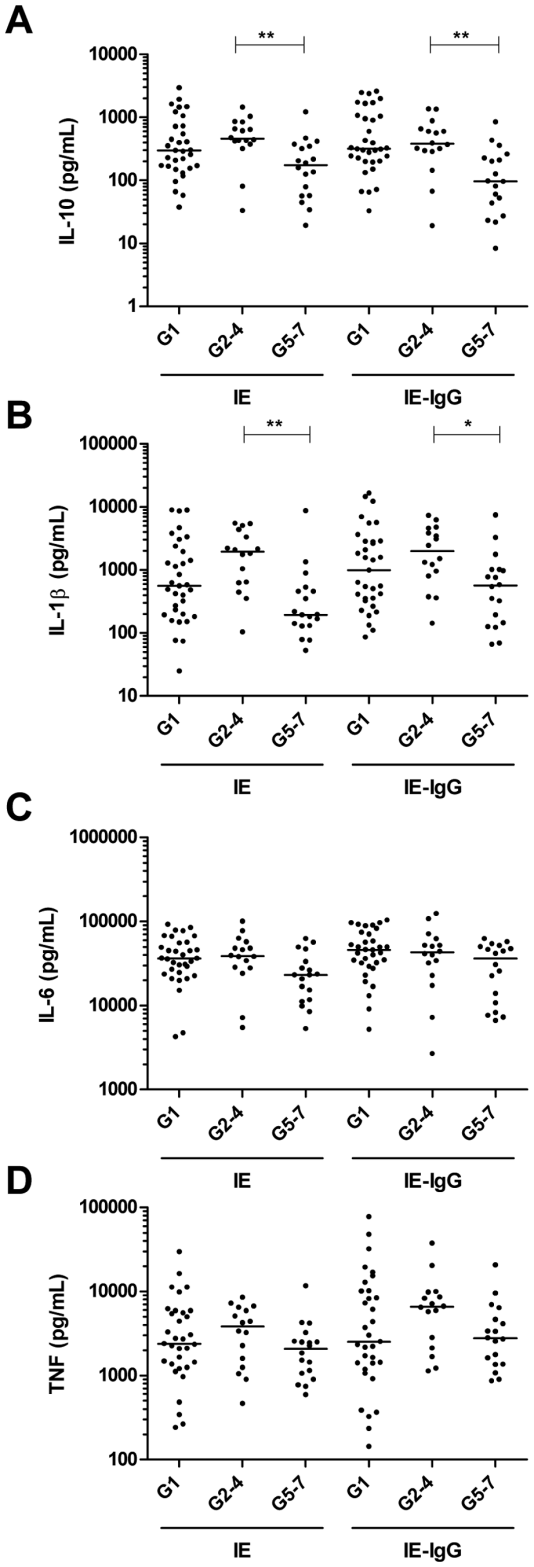
PBMC derived from G5–7 women secrete lower levels of IL-10, IL-1β and IL-6. PBMC (2×10^5^/well) were exposed to 6×10^5^ unopsonised IE or IE opsonised with 9% HI autologous plasma (IE-IgG). After 48 h, culture supernatants were quantified for (**A**) IL-10, (**B**) IL-1β, (**C**) IL-6 and (D) TNF using a commercial multi-plex assay. Cytokine production from resting PMBC was subtracted from IE induced cytokine to eliminate the contribution of constitutive cytokine production and data is presented on a log_10_ scale. Comparison between three groups (G1, G2–4, G5–7) was achieved using a non-parametric One Way ANOVA: Kruskal-Wallis test and the Dunn's Multiple Comparison post-test. * p<0.05, ** p<0.01.

Less IL-10 was detected in G5–7 compared to G2–4 PBMC in response to IE (174 (57–355) versus 459 (387–796) pg/mL; p<0.01) and plasma-opsonised IE (97 (40–236) versus 382 (290–646) pg/mL; p<0.01) (Figure 2A). IL-10 levels were also significantly lower in PBMC derived from G5–7 women compared to G1 in response to plasma-opsonised IE (97 (40–236) versus 317 (201–1058) pg/mL; p<0.01). In summary, PBMC obtained from G5–7 secrete significantly lower levels of the Th-2 immunoregulatory cytokine IL-10 than those in the G1 and G2–4 groups when exposed to IE. Less pro-inflammatory IL-1β (Figure 2B) was secreted by G5–7 compared to G2–4 PBMC for both unopsonised (195 (131–479) versus 1,964 (629–4,127) pg/mL; p<0.01) and plasma-opsonised IE (567 (142–1,015) versus 1993 (843–4,409) pg/mL; p<0.05). Less pro-inflammatory IL-6 (Figure 2C) was secreted by G5–7 compared to G1 PBMC in response to unopsonised IE (23,076 (11,610–36,809) versus 36,500 (24,127–56,120) pg/mL; p<0.01). There was also a trend towards lower TNF secretion detected in G5–7 compared to G2–4 PBMC for both unopsonised and plasma-opsonised IE (Figure 2D). Overall, there was decreased IL-10, IL-1β and IL-6 secretion and a trend towards lower secretion of TNF in G5–7 compared to G2–4 women; for IL-10 and Il-6, G5–7 women's PBMC also produced less cytokine than cells from G1 women in response to opsonised or unopsonised IE.

### PBMC derived from G5–7 secrete higher levels of CXCL8, CCL8, IFNγ and CXCL10

**Figure 3:**
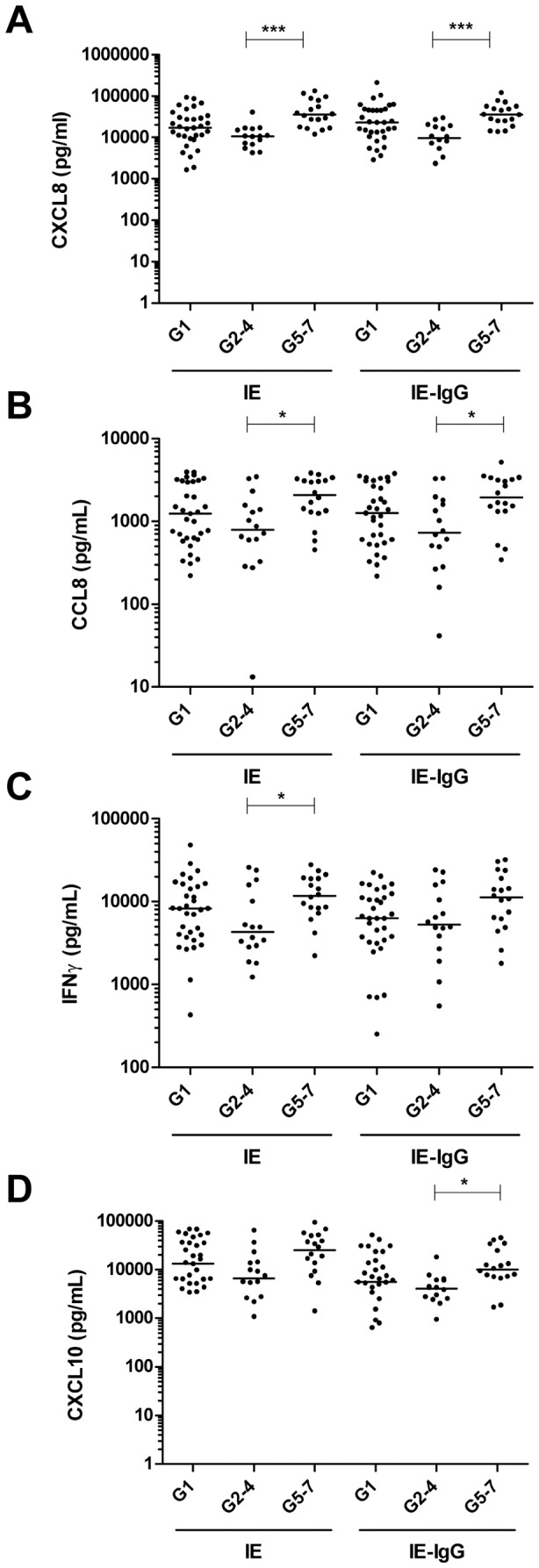
PBMC derived from G5–7 secrete higher levels of CXCL8, CCL8, IFNγ and CXCL10. PBMC (2×10^5^/well) were exposed to 6×10^5^ unopsonised IE or IE opsonised with 9% HI autologous plasma (IE-IgG). After 48 h, culture supernatants were quantified for (**A**) CXCL8, (**B**) CCL8, (**C**) IFNγ and (**D**) CXCL10. Both CXCL8 and CCL8 were quantified using ELISA while IFNγ and CXCL10 were quantified using a commercial multi-plex assay. Cytokine production from resting PBMC was subtracted from IE induced cytokine to eliminate the contribution of constitutive cytokine production and data is presented on a log_10_ scale. Comparison between three groups (G1, G2–4, G5–7) was achieved using a non-parametric One Way ANOVA: Kruskal-Wallis test and the Dunn's Multiple Comparison post-test. * p<0.05, *** p<0.001.

In contrast to IL-10, IL-1β and TNF, there was more CXCL8 secreted by G5–7 compared to G2–4 PBMC in response to IE (35,516 (17,598–81,278) versus 10,637 (5,783–15,957) pg/mL; p<0.001) and plasma-opsonised IE (35,851 (23,625–56,523) versus 9,657 (3,877–18,824) pg/mL; p<0.001) (Figure 3A). Secretion of CXCL8 was also significantly higher in PBMC derived from G5–7 compared to G1 in response to unopsonised IE (35,516 (17,598–81,278) versus 17,000 (9,130–32,024) pg/mL; p<0.05). In addition, CXCL8 levels were lower in PBMC derived from G2–4 compared to G1 in response to plasma-opsonised IE (9,657 (3,877–18,824) versus 22,910 (10,293–47,188) pg/mL; p<0.01). Therefore, IE-stimulated PBMC obtained from G5–7 secrete significantly higher levels of the chemotactic factor CXCL8 than those in the G2–4 groups. Following a similar pattern, levels of CCL8 (Figure 3B) were higher in PBMC from G5–7 women compared to G2–4 in response to unopsonised IE (2,087 (1,286–3,160) versus 795 (394–1528) pg/mL; p<0.05) and plasma-opsonised IE (1,945 (1,329–3,237) versus 732 (335–1753) pg/mL; p<0.05). Higher IFNγ levels (Figure 3C) were detected in G5–7 compared to G2–4 PBMC for unopsonised IE only (11,714 (8,058–19,087) versus 4,306 (2,852–14,456) pg/mL; p<0.05). More CXCL10 (Figure 3D) was detected in G5–7 compared to G2–4 PBMC for plasma-opsonised IE only (10,062 (7,089–27,157) versus 4,071 (2,477–6,160) pg/mL; p<0.05).

### Levels of IL-1Ra and CCL3 elicited from PBMC did not vary with gravidit

**Figure 4:**
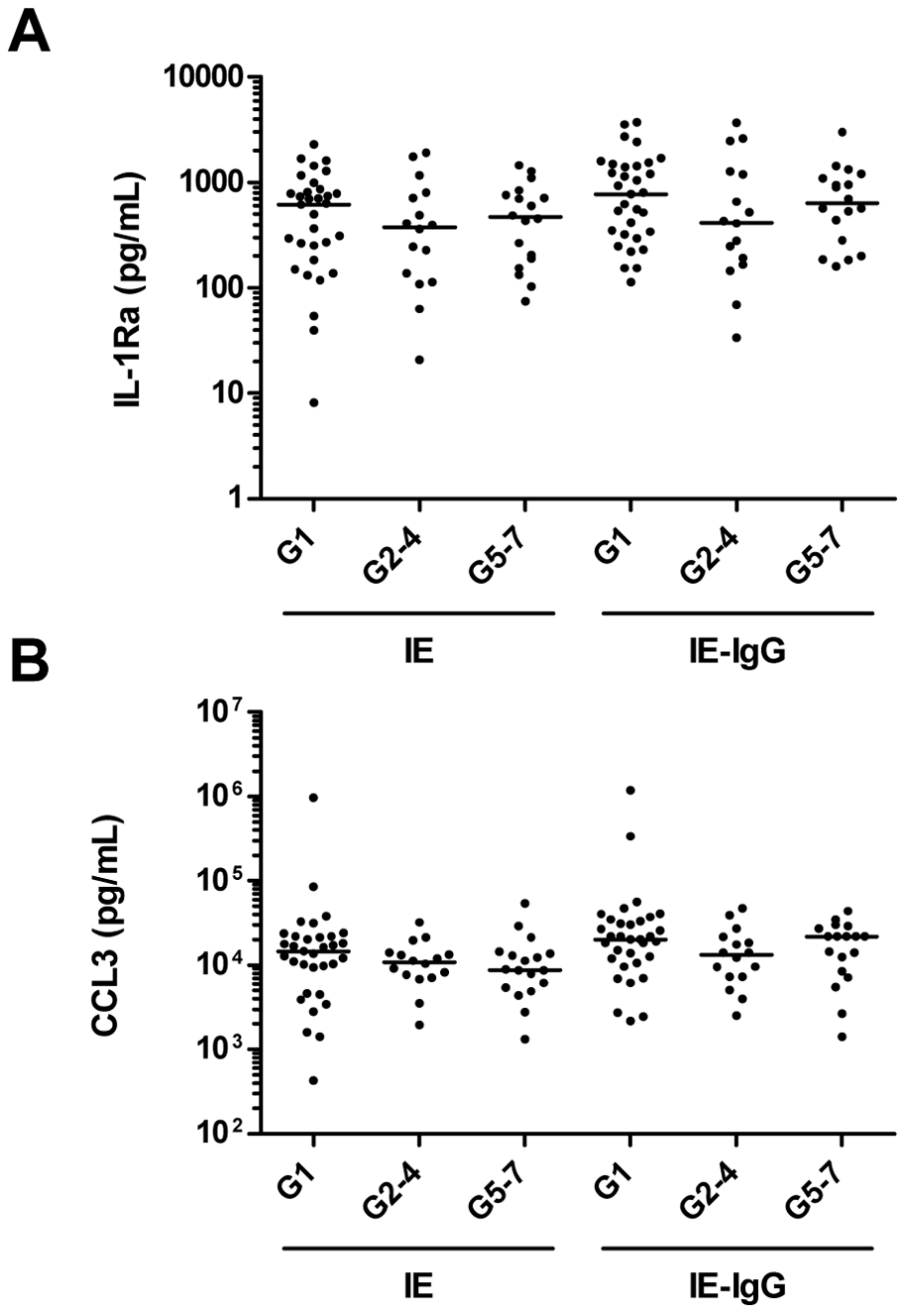
Levels of IL-1Ra and CCL3 released by PBMC did not vary with gravidity. PBMC (2×10^5^/well) obtained from primigravid and multigravid women were exposed to 6×10^5^ unopsonised IE or IE opsonised with 9% HI autologous plasma (IE-IgG). After 48 h, culture supernatants were quantified for (**A**) IL-1Ra and (**B**) CCL3 using a commercial multi-plex assay. Cytokine production from resting PBMC was subtracted from IE induced cytokine to eliminate the contribution of constitutive cytokine production and data is presented on a log_10_ scale. Comparison between three groups (G1, G2–4, G5–7) was achieved using a non-parametric One Way ANOVA: Kruskal-Wallis test and the Dunn's Multiple Comparison post-test.

No variations in the level of IL-1Ra and CCL3 secretion were observed between women in the 3 groups (Figure 4).

Our results demonstrate that PBMC from grand multigravidae are characterised by a distinct cytokine and chemokine response to pregnancy-specific IE.

## Discussion

This study explored the role of gravidity in modifying the early cellular immune response of pregnant women to *P. falciparum* IE. Our methodology examined cytokine and chemokine secretion from PBMC derived from malaria exposed pregnant women without current malaria infection by microscopy. Thus we investigated potential immune responses, and how gravidity influences these, and not the actual response to current malaria infection. The majority of studies conducted to investigate changes in cytokine and chemokine secretion caused by placental malaria or associated with increasing gravidity involve measuring circulating peripheral or placental plasma levels, mRNA expression using PCR, or protein levels by histological techniques using fixed placental tissue. The major limitations to these approaches are that plasma cytokine levels may not reflect the ability of peripheral blood cells to secrete cytokines in response to infection, and that measurements taken during acute infection provide limited information about protective responses, as participants are already infected. Our study used an *in vitro* approach to investigate the capacity of circulating PBMCs derived from women without current malaria infection to respond to CS2 IE expressing VAR2CSA, therefore reflecting baseline cellular responses in otherwise healthy uninfected women. It would be of interest to compare responses in individuals with current malaria infection (detected by microscopy, or by PCR). A low prevalence of malaria in our study cohort precluded this comparison. One limitation of our model was that peripheral PBMC may not be fully representative of those found in the placenta, and future studies of placental blood cells are indicated. Limited PBMC yield prevented us from identifying the cytokine-and chemokine-producing populations in these women. In children from PNG, most of the early IFNγ response to unopsonised IE is from γδ or αβ T cells [25], and TNF and IL-6 are derived mostly from monocytes and γδ T cells [26]. The interactions between IE and host leukocytes are also of interest. After 48 h incubation IE will have been ingested or killed, releasing parasite products including hemozoin and glycosylphosphatidylinositol, both of which can stimulate inflammatory cytokine secretion [27], [28], but VAR2CSA expression on IE may modify cytokine responses [29]. Phagocytic uptake of opsonised IE by purified macrophages leads to marked induction of TNF, IL-1β and IL-6 [24]. The differences we observe in this study between G2–4 and G5–7 women's responses may reflect a shift from a more marked macrophage inflammatory response to a greater role for IFNγ-mediated responses in women with a greater history of exposure to malaria in pregnancy.

The effects of modulation of the immune response by the syncytiotrophoblast [30] were not taken into account. HIV burden was unlikely to impact our study as a recent audit at the antenatal clinic at Modilon Hospital from November 2009 to August 2012 revealed 1.09% of study participants were infected (3142 women tested, Unger, HW, Bolnga JW; unpublished data). Study participants were not tested for helminth infections, which are common in PNG [31]. Such infections may have effects on malaria immunity [32], potentially modifying the susceptibility and immune response to malaria antigens. As expected, G1 (primigravid) women were significantly younger than other women; studies with larger sample sizes could adjust for this possible confounding factor.

This study identified significant gravidity-dependent differences in the pattern of cytokine production in response to CS2 IE. PBMC derived from grand multigravidae secreted significantly lower levels of IL-10, IL-1β and IL-6, and somewhat lower amounts of TNF than cells from women in their second to fourth pregnancy. By contrast, PBMC derived from grand multigravidae secreted higher levels of CXCL8, CCL8, IFNγ and CXCL10 compared to women in their second to fourth pregnancy in response to IE. For most cytokines and chemokines, levels secreted by G5–7 women were similar to those from G1. This observation is consistent with the high susceptibility to placental malaria of women in their first pregnancy [1], and observations suggesting that grand multigravidae show greater responses to malaria prevention than G2–4 women [10]. Thus in G2–4, high secretion of IL-10, IL-1β and TNF but low secretion of CXCL8, CCL8, IFNγ and CXCL10 may confer a favourable outcome.

Higher levels of opsonising antibody to parasites expressing VAR2CSA were present in plasma derived from G2–4 and G5–7 women compared to G1 providing further evidence for the role of antibody-mediated immunity in modulating the prevalence and intensity of malaria infection in successive pregnancies. This result is in agreement with previous studies published in our laboratory showing IgG opsonic activity in serum is gravidity dependent in pregnant women in Malawi [23], [33]. In the present study we compared cytokine responses from PBMC exposed to unopsonised and opsonised IE, based on our previous observation that IE opsonisation results in increased macrophage-derived pro-inflammatory cytokine secretion [24]. Opsonisation resulted in increases in cytokine and chemokine secretion using this PBMC model, consistent with our previous work, although the magnitude of the increase associated with opsonisation was less than when using macrophages. Our observations suggest that antibody is a main driver of differences between PG and higher gravidities. No difference in opsonic antibody levels between G2–4 and G5–7 were observed indicating the differences in cytokine and chemokine secretion between these two groups were due to cellular immune responses not humoral immunity.

PBMC obtained from G5–7 secreted significantly lower levels of the key immunoregulatory Th-2 cytokine IL-10 compared to primigravidae and G2–4. Both decreased [3] [34] and increased [16], [35], [36] IL-10 levels have been reported in maternal malaria conferring immunopathology or protection. Consistent with a general modulation of immune responses with increasing gravidity, there was also less secretion of IL-1β and TNF by PBMC from G5–7 women compared to G2–4 women. Significantly increased IL-1β transcript levels [15] and increased secretion of TNF have been associated with placental malaria and (for TNF) with a heightened risk of severe anaemia and LBW [3], [17]. Lower production of both IL-10 and inflammatory cytokines by PBMC from G5–7 women could indicate a maturation in the innate immune responses in women of higher gravidity, possibly driven by acquired immune cells.

Changes in IFNγ with placental malaria vary by study [14], [16], [17], [37] but it clearly has an important role in successful pregnancy outcome [38]. We observed increased IFNγ in G5–7 compared to G2–4 using unopsonised IE and similar, non-significant trend with opsonised IE. Parasite-induced early IFNγ originates from γδ T cells and αβ T cells in semi-immune PNG children [25]. Further work is needed to investigate the cellular source of IFNγ which was impracticable in the present study due to limited PBMC yield. CXCL10 was also higher in G5–7 exposed to plasma-opsonised IE. CXCL10 is known to be induced by IFNγ [39] and has chemotactic activity for activated Th-1 lymphocytes. The increased activity of this axis with gravidity suggests its potential importance in immunity to *P. falciparum* malaria.

Increased chemokine production by monocytes and macrophages may be an important trigger for accumulation of monocytes, neutrophils and other cells in the placenta leading to increased phagocytosis and parasite clearance. Levels of the neutrophil chemotactic factor CXCL8 were higher in G5–7 compared to G2–4 suggesting increased potential for chemotaxis and phagocytosis of IE, which might limit malaria infection. Placental malaria infection has previously been associated with elevated placental CXCL8 mRNA expression and plasma concentrations; the latter were also associated with placental monocyte infiltration [13]. These results and our finding of increased CXCL8 in G5–7 may explain the increased numbers of neutrophils in placental malaria [40]. Levels of CCL8, which is chemotactic for monocytes, T lymphocytes and NK cells, and is induced by IFNγ [41], were also higher in G5–7 compared to G2–4.

Among other chemokines measured, high levels of CCL3 secretion were detected, which did not differ with gravidity. Previous studies have demonstrated that placental malaria infection was associated with elevated CCL3 mRNA levels which correlated with monocyte density in the placental intervillous space [13], and that cultured maternal intervillous leukocytes from infected placentas secrete higher levels of CCL3 than leukocytes from uninfected women [16]. Syncytiotrophoblast cells stimulated with LPS produce CCL3 indicating these cells are also an important source of this chemokine in the placenta [42].

Our results demonstrate that PBMC from grand multigravidae are characterised by a distinct cytokine and chemokine response to pregnancy-specific IE. Compared to cells from gravida 2–4 women, they showed decreased production of pro-inflammatory cytokines IL-1β, IL-6 and TNF and the regulatory cytokine IL-10, and increased production of IFNγ and the downstream protein CXCL10, and of chemokines CXCL8 and CCL8. In combination with the acquisition of pregnancy-specific opsonising antibodies, which are rare in first pregnancy, these findings are more in keeping with an increased maturation of the immune response in grand multigravidae. The chemokine and cytokine responses suggest early engagement of T cells and NK cells (and possibly neutrophils) in the response to IE, rather than marked inflammatory macrophage responses, which are seen in the placenta in early pregnancies. Rather than indicating susceptibility to placental infection or its consequences, these responses indicate that grand multigravidae display a distinct peripheral cellular response with counter-regulatory and feedback mechanisms in place.
